# Electrochemical sensing platform based on screen-printed carbon electrode modified with plasma polymerized acrylonitrile nanofilms for determination of bupropion

**DOI:** 10.1007/s00604-023-05971-0

**Published:** 2023-09-14

**Authors:** Maria Madej, Agata Trzcińska, Justyna Lipińska, Ryszard Kapica, Maciej Fronczak, Radosław Porada, Jolanta Kochana, Bogusław Baś, Jacek Tyczkowski

**Affiliations:** 1 Faculty of Chemistry, Department of Analytical Chemistry, Jagiellonian University, Gronostajowa 2, 30-387 Kraków, Poland; 2 Faculty of Materials and Ceramics, Department of Analytical Chemistry and Biochemistry, AGH University of Science and Technology, A. Mickiewicza 30, 30-059 Kraków, Poland; 3 Faculty of Process and Environmental Engineering, Department of Molecular Engineering, Lodz University of Technology, Wólczańska 213, 93-005, Lodz, Poland

**Keywords:** Modified electrodes, Plasma polymerized acrylonitrile, Screen-printed electrodes, Cold plasma deposition (PECVD), Bupropion, Voltammetry

## Abstract

A original electrochemical sensing platform, based on screen-printed electrodes modification with plasma polymerized acrylonitrile (pp-AN) nanofilms is proposed. For that purpose, plasma-enhanced chemical vapor deposition (PECVD) process was conducted in a parallel plate (13.56 MHz) plasma reactor for 2 min with discharge power of 10 W. The surface topography and electrochemical properties of prepared sensors were investigated by X-ray photoelectron spectroscopy, scanning electron microscopy, energy dispersion spectroscopy, electrochemical impedance spectroscopy, and cyclic voltammetry. The electrochemical characteristics of pp-AN/SPCE and pp-AN/SPAuE sensors was investigated for model redox pair [Fe(CN)_6_]^4−/3−^. Conducted research confirmed the excellent chemical stability, durability, wide potential window, high signal-to-noise (S/N) ratio, and, most importantly, the ability to standardize the sensors. The pp-AN/SPCE sensor was applied to the determination of bupropion, an antidepressant drug whose intake has increased dramatically during the COVID-19 pandemic. The voltammetric response of pp-AN/SPCE for BUP was linear in two concentration ranges of 0.63–10.0 and 10.0–50.0 μmol L^−1^, with a detection limit of 0.21 μmol L^−1^. Satisfactory recoveries (96.2–102%) and good precision (RSD below 4.1%) obtained for environmental and biological samples confirmed the usefulness of the sensor for the analysis of various kinds of samples.

**Graphical Abstract:**

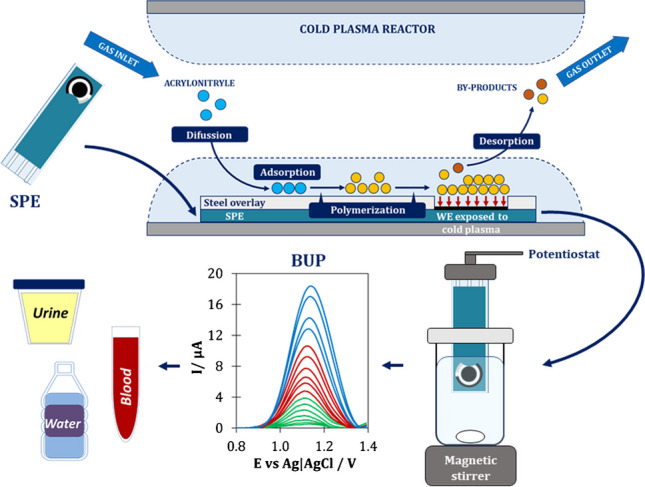

**Supplementary Information:**

The online version contains supplementary material available at 10.1007/s00604-023-05971-0.

## Introduction

Modern chemical analysis uses many tools that enable the detection and quantification of a wide range of chemical compounds in samples with a variety of matrixes. Works on new chemical sensors, especially electrochemical ones, are a particularly intensively explored area of research. In order to provide the required stability, durability, selectivity, sensitivity, and accuracy of sensors, new, often functionalized electrode materials are designed, as well as novel methods of physical or chemical modification of substrate electrode surface are developed. In this aspect, the nanofilms obtained in the chemical vapor deposition process (CVD) are a particularly promising group of functional materials that could replace conventional materials used for electrode modification like carbon nanostructures or metal nanoparticles [1].

The CVD process allows for a production of thin films as a result of a chemical reaction taking place on a heated substrate in the vacuum chamber, where vapors of a selected compound are supplied, the so-called precursor. In contrast to the physical methods of vapor deposition, such as evaporation and sputtering, the CVD technique ensures high homogeneity of the produced films with a precisely defined structure, and at the same time enables strict control of growth of the deposited film by adjusting such experimental parameters as the temperature, pressure, deposition time, precursor concentration, and type of carrier gas [2]. A particularly interesting type of CVD technique is plasma-enhanced chemical vapor deposition (PECVD), which allows to carry out the deposition process at room temperature. In this case, plasma (high-energy, ionized gas) acts as an energy source that induces the decomposition of the precursor into active species (radicals, ions, etc.). Depending on the used precursor, obtained nanofilms may show very good electrical and catalytic properties, as well as a high surface area, with ensured uniformity of the film and its high mechanical durability [3, 4].

The modification of electrochemical sensors by plasma treatment is a relatively new approach and only few works dealing with this topic can be found in the literature. Wardak et al. developed a dopamine sensor based on laccase deposition on the surface of a glassy carbon electrode in corona discharge plasma reactor [5]. The treatment of ion-selective electrodes for nitrite detection with cold plasma was investigated by S. Sedaghat [6]. We also described the first example of deposition of plasma polymerized nanofilms directly at the surface of screen-printed electrodes [7].

Bupropion (BUP), a monocyclic aminoketone that belongs to the class of substituted cathinones, is a commonly used atypical antidepressant, which acts as a dual norepinephrine and dopamine reuptake inhibitor as well as nicotinic receptor antagonist. Due to its specific pharmacological action, BUP is not only used in treatment of major depressive disorder but also for reduction of nicotine carvings and ADHD symptoms. Noteworthy, BUP is considered an only effective drug against seasonal depression. In addition, BUP is much more effective than selective serotonin reuptake inhibitors at improving symptoms of hypersomnia and fatigue. Despite numerous advantages, it is worth noting that the use of BUP carries a much higher risk of epileptic attacks. BUP is effectively metabolized in the liver to three active metabolites, i.e., hydroxybupropion, threohydrobupropion, and erythrohydrobupropion. Only 0.5% of the administered dose is excreted unchanged in the urine [8, 9].

Mainly chromatographic and spectrometric methods have been developed for BUP determination in pharmaceutical and biological samples, i.e., high-performance liquid chromatography with a spectrofluorometric detection [10], liquid chromatography coupled with tandem mass spectrometry [11], or capillary electrophoresis combined with phosphorescence detection [12]. In the case of electroanalytical methods, only a few examples of electrochemical sensors enabling the determination of BUP have been reported so far, including ion-selective PCV membrane electrodes [13], dropping mercury electrode [14], bulk glassy carbon electrode [15], and a carbon screen-printed electrode modified with molecularly imprinted polymer, gold nanoparticles, and graphene oxide as an electrochemical impedance spectroscopy sensor [16].

The paper presents for the first time an original electrochemical sensing platform, which was produced by depositing a plasma polymerized acrylonitrile (pp-AN) nanofilm on the surface of a screen-printed electrode (SPE) using plasma-enhanced chemical vapor deposition technology. The use of the PECVD process for SPE modification creates a unique opportunity for the standardization of manufactured sensors. Standardization is essential for serial production; without it, each sensor is just an individual device with different metrological and operational parameters. The successful application of the pp-AN/SPCE sensor to detect and determine BUP proves its effectiveness and attractiveness.

## Experimental

### Apparatus

The plasma polymerized nanofilms were deposited onto SPCEs (Dropsens C110, Metrohm) or SPAuEs (Dropsens C220AT, Methrom) in a parallel plate radio frequency (13.56 MHz) plasma reactor operating at low pressure, analogous to that shown in [4]. For voltammetric measurements, a bulk or modified SPEs were connected with a CAC (Metrohm) cable connector to the M161 multipurpose electrochemical analyzer equipped with the M164 electrode stand (both mtm-anko, Poland). The impedance spectra were recorded using the μAUTOLAB III analyzer (EcoChemie, the Netherlands) with NOVA 2.0 software. The surface morphology of SPEs was examined using a Quanta 200F (FEI Company) and Vega3 (TESCAN) scanning electron microscopes coupled with X-ray energy-dispersive spectroscopy (EDS) detector. The chemical composition and molecular structure of the surface of the SPEs and nanofilms deposited on these electrodes were investigated by X-ray photoelectron spectroscopy (XPS), using an AXIS Ultra spectrometer (Kratos Analytical Ltd., UK). Monochromatic Al Kα X-rays (1486.6 eV) were used for the analysis. The spectra were acquired from three different spots (300 × 700 μm^2^). The anode power was set at 180 W and the hemispherical electron energy analyzer worked at a pass energy of 20 eV for all the high-resolution measurements.

### Chemicals

Acrylonitrile (Sigma-Aldrich) was used as a precursor for the plasma deposition process. For the preparation of 10 mmol L^−1^ BUP standard solution, the appropriate amount of a bupropion hydrochloride (Merck) was weighted and dissolved in 2 mL of ultrapure water, and then stored in a temperature of 4 °C. The standard solution of [Fe(CN)_6_]^4−/3−^ was prepared by mixing equal volumes of 0.4 mol L^−1^ potassium hexacyanoferrate(II) and potassium hexacyanoferrate(III) solutions (both purchased from Chempur). Potassium chloride, sodium nitrate, sodium dihydrogen phosphate, and hydrogen phosphate disodium were obtained from Chempur. For interference study, the following substances were used: sodium nitrate (Chempur), glucose (Micropharm), humic acid, lactose, magnesium sulfate, magnesium stearate, talc, titanium dioxide, starch, Triton X-100, cetrimonium bromide, and sodium dodecyl sulfate (Sigma-Aldrich). The precision and accuracy was verified on the basis of the BUP quantification in the certified reference materials (CRMs) of Waste Water SPS-WW2 Batch no. 113 (Spectrapure Standards AS), Ground Water ES-H-2 EnviroMAT (SCP Science), synthetic urine Seronorm Trace Elements Urine (Sero), and synthetic serum Nortrol (Thermo Scientific) enriched with an analyte. The Vistula river water sample was collected in Kraków, subjected to filtration and then stored in a fridge for analysis. All aqueous solutions were prepared with reagents of analytical grade purity and ultrapure water from the HLP 5 system (Hydrolab).

### Plasma deposition of polymer nanofilms

The deposition of pp-AN nanofilms was performed analogously as it was described in our previous short communication [7]. The SPEs were placed in reactor chamber and covered with customized steel overlays. After closing the reactor, the vacuum was allowed to establish to pressure below 10^−1^ Pa, and then the electrode surface was etched with argon plasma for 1 min at the power of 100 W (with Ar flow rate of 2.0 sccm). After etching, acrylonitrile vapor was supplied to the reactor chamber and plasma with power of 10 W for 2 min was initiated. When the deposition process was completed, the electrodes were left for 10 min in the reactor chamber to prevent the damage of pp-AN films due to drastic pressure changes. Electrodes prepared in this way were stored at room temperature without access to light up to their use.

### Electrochemical measurements

The cyclic voltammetry (CV) measurements were conducted in the presence of [Fe(CN)_6_]^4−/3−^ as a reversible redox system in 0.1 mol L^−1^ solution of KCl, with a scan rate of 0.1 V s^−1^ and potential range between −0.4 and 0.8 V for SPCE or −0.1 and 0.5 V for SPAuE. Preliminary experiments performed for BUP were carried out in the phosphate buffer solution with pH 7.0 and concentration of 0.1 mol L^−1^ using CV with a scan rate of 0.05 V s^−1^. After method optimization, the BUP quantification was conducted using staircase voltammetry (SCV) in ammonium buffer (pH 8.0) with a potential step of 8 mV, step width of 80 ms, accumulation potential of 100 mV, and accumulation time of 120 s. For each mean value, the confidence interval was calculated based on following formula:

$$CI={t}_{\alpha, n-1}\bullet \frac{s}{\sqrt{n}}$$

where *t*_*α*, *n* − 1_ is the critical value of Student’s *t* distribution for significance level α = 0.05 and (*n* −1) degrees of freedom, *s* is the standard deviation, and *n* is the number of measurements.

The electrochemical impedance spectroscopy (EIS) measurements were performed in the frequency range of 100 kHz–25 mHz with an amplitude of 10 mV at the formal potential of the redox pair [Fe(CN)_6_]^4−/3−^ at concentration of 0.2 mmol L^−1^ in 0.1 mol L^−1^ KCl. To obtain the key figures of merit, the equivalent electrical circuit was fitted to each impedance spectrum.

### Samples analysis

The sample of environmental water was collected from Vistula river in Krakow, filtered and stored in 4 °C before analysis. Due to the fact that BUP was not detected in the tested sample, river water, as well as CRMs of wastewater, groundwater, synthetic urine, and serum were spiked with the analyte for the recovery tests. Considering the matrix composition of each sample, the enriched samples were diluted as follows: river water 3-fold, CRMs of wastewater and groundwater 15-fold, urine 100-fold, and blood serum 200-fold. Then BUP quantification was conducted using standard addition method.

## Results and discussion

### Selection of plasma polymerization conditions

The choice of electrode surface modification conditions is crucial to provide high sensitivity, durability, and stability of the sensor performance. Apart from the chemical structure of the precursor itself, the properties of films produced in the PECVD process are affected by various factors, like the discharge power (*P*_*w*_), the deposition time (*t*_*d*_), or the composition of the carrier gas supplying the precursor to the plasma reactor chamber. Based on preliminary studies on the direct deposition of pp-AN nanofilms on the surface of SPEs, it was proved that the better properties in terms of electrochemical applications exhibit films deposited at lower discharge power and short deposition times. The application of low discharge power enables strict control of deposition rate, which gives a possibility to prepare sensors with diverse properties [7].

At initial stage of research, the various precursors (acrylonitrile, diethoxydimethylsilane, triethoxymethylsilane and 1,1,3,3-tetramethyldisiloxane) were investigated in terms of its conductive properties. For all these tested precursors, a decrease of the DC electrical conductivity measured across the deposited films with the increasing *P*_*w*_ and *t*_*d*_ was observed, which is consistent with our previous experiments. Among tested precursors, acrylonitrile exhibits the highest vapor pressure, which allows for carrying out the deposition process even at a *P*_*w*_ of 10 W. Thus, it is possible to produce films with thickness not exceeding 100 nm. The remaining compounds are characterized by significantly lower vapor pressures compared to AN. Therefore, creating sufficient pressure to initiate the plasma in the reactor requires a *P*_*w*_ at least equal to 20 W. It is also worth noting that for triethoxymethylsilane and 1,1,3,3-tetramethyldisiloxane, it was necessary to supply inert carrier gas (Ar) to the reactor chamber in order to achieve the appropriate pressure to initiate the plasma even at *P*_*w*_ of 20 W. Based on the conducted research, it can be concluded that plasma polymerized pp-AN nanofilms deposited at a *P*_*w*_ of 10 W and for 2 min have the greatest potential for the electrochemical performance of sensors. Thus, in order to verify this hypothesis, a study of surface topography and electrochemical experiments were conducted for SPCE and SPAuE modified with pp-AN nanofilms.

### Characterization of screen-printed electrodes modified with plasma polymerized nanofilms

**Fig. 1:**
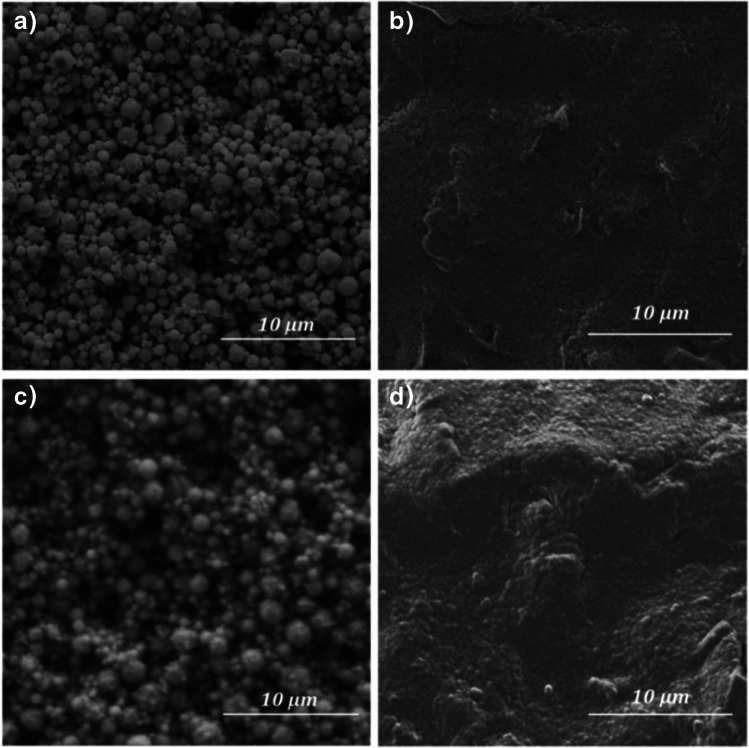
SEM images of SPAuE and SPCE surface before (**a**, **b**) and after deposition of the pp-AN film (**c**, **d**)

In order to investigate the morphology of modified electrodes, the surface of gold and carbon electrode before and after deposition of pp-AN film were characterized by SEM (Fig. 1). Obtained images showed that the bare SPAuE and SPCE differ significantly in their surface morphology, which is justified by different production technology. The gold electrode is characterized by high roughness, while the carbon electrode has a nearly smooth surface with slight irregularities. After pp-AN deposition, both SPCE and SPAuE were covered with a homogeneous, continuous film reflecting the topography of their surfaces.

Based on the EDS analysis and recorded XPS spectra, the elemental composition of the surface of both tested sensors was examined. The EDS mapping of the Au electrode modified with pp-AN (Fig. S1) revealed the uniform distribution of carbon atoms, and thus also the pp-AN nanofilm on the SPAuE. Closer analysis showed that few spots at Au surface were not covered with pp-AN, which suggest that the produced film can be discontinuous at points of the highest surface roughness. Consequently, on the surface of the pp-AN/SPAuE, bulk spots of Au could be present, which may affect the electrochemical properties of such sensor.

In the case of the carbon electrode, the changes in elemental composition observed in both EDS and XPS measurements (Tab. S1) confirmed the presence of deposited nanofilm on the surface of SPCE by revealing the appearance of nitrogen after the deposition of pp-AN. It should be added that the differences in the concentration of elements determined by the XPS and EDS analysis are understandable because it results from differences in the thickness of the analyzed film material: for XPS, it is a surface layer with a thickness of single nanometers; in EDS measurements, we look much deeper into the film structure. A significantly higher concentration of oxygen in the XPS compared to the EDS measurements proves, in this case, a distinct oxidation of the surface of both the bare SPCE electrode and the surface of the deposited pp-AN film.

**Fig. 2:**
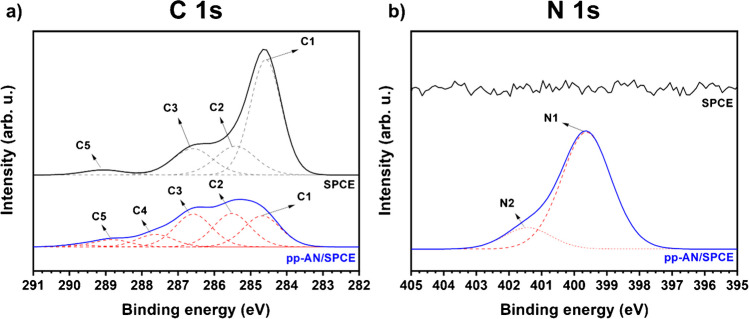
XPS spectra for SPCE and pp-AN/SPCE samples with detailed comparison for C 1s (**a**) and N 1s (**b**) spectral regions.

The analysis of narrow XPS spectra for the regions corresponding to C1s (282–291 eV) and N1s (395–405 eV) showed at Fig. 2, confirmed the change in the molecular structure of the SPCE surface after pp-AN deposition (*for more details*, *see* *Supplementary Material*) [17–22]. The plasma deposition of acrylonitrile on the SPCE resulted in the formation of a pp-AN nanofilm on its surface containing carbon and nitrogen, as well as oxygen that originates from the reaction between radical states formed in the plasma deposition process and oxygen and water molecules present in the air atmosphere. The molecular structure of this film, according to XPS studies, is very complex, beginning with simple fragments of polymerized acrylonitrile chains and ending with condensed rings with graphite nitrogen.

### Electrochemical performance of pp-AN/SPCE

**Fig. 3:**
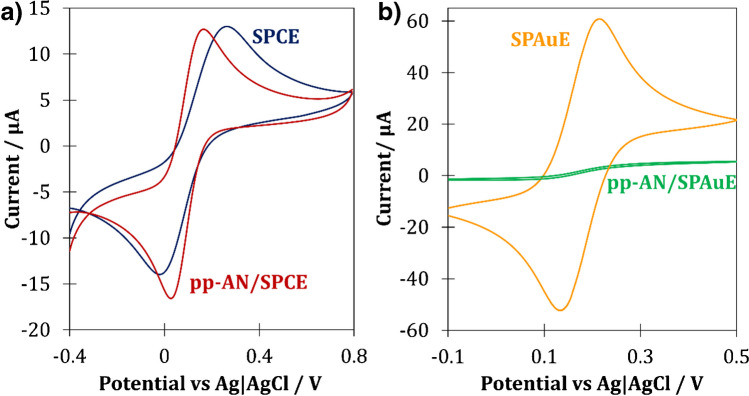
CV voltammograms recorded in 0.1 mol L^−1^ KCl containing 1.0 mmol L^−1^ of [Fe(CN)_6_]^4−/3−^ with a use of SPCE (**a**) and SPAuE (**b**) before surface modification and with deposited pp-AN nanofilm (*ν* = 0.1 V s^−1^)

For the evaluation of electrochemical behavior of SPCE and SPAuE modified with pp-AN nanofilms, CV measurements for the reversible redox couple of [Fe(CN)_6_]^4−/3−^ were performed. Cyclic voltammograms recorded before and after modification of the SPCE surface with pp-AN nanofilm showed similar features (Fig. 3a). In both cases, well-defined anodic and cathodic peaks were observed. However, the pp-AN/SPCE is characterized by higher sensitivity (ca. 30%), more symmetrical anodic and cathodic peaks (with *I*_p,ox_/*I*_p,red_ ≈ 1) and peak separation (Δ*E*) of 163 mV, while for unmodified SPCE *ΔE* higher than 200 mV was obtained. The improvement of electrochemical performance of pp-AN/SPCE compared to bare SPCE may result not only from facilitating the electron transfer but also the development of electrochemically active surface area of the electrode. Previous studies revealed that the pp-AN/SPCE surface area was about 30% larger than for unmodified electrode, which were equal to 0.131 cm^2^ and 0.104 cm^2^, respectively [7].

In the case of SPAuE and pp-AN/SPAuE, the drastic change in the shape of recorded cyclic voltammograms was observed (Fig. 3b). For bulk SPAuE, well-defined, symmetrical oxidation and reduction peaks were observed, while for pp-AN/SPAuE recorded voltammograms took the sigmoidal shape, which suggest the presence of so-called microelectrode effect. In that case, the macroelectrode behaves like the set of interconnected microelectrodes [23]. This rare phenomenon most probably results from a surface topography of the SPAuE and the discontinuity of pp-AN film on its surface.

In order to assess the suitability of the tested sensors for the determination of BUP, cyclic voltammograms using bulk and modified SPEs were recorded. The oxidation potential of secondary amine derivatives in neutral media is usually observed at ca. 1.0 V [24]. Thus, the behavior of tested sensors while extending potential window in the anodic direction was examined first, in order to investigate the durability of pp-AN film in wider potential window and to indicate the range of useful potentials for each sensor. No peaks indicating the course of redox processes were observed on the recorded voltammograms, which confirmed the good stability of the pp-AN film in the tested potential range (*data not shown*). In the case of SPAuE and pp-AN/SPAuE already at the potential of 0.8 V, a rapid increase in the background current caused by Au oxidation was observed (*data not shown*). Therefore, focusing on the BUP electroanalysis, further tests of gold electrodes were discontinued.

For closer investigation of the pp-AN nanofilm influence on the metrological parameters of electrode, the EIS was employed. The Nyquist plots were recorded using SPCE before and after deposition of pp-AN in the presence of 0.2 mmol L^−1^ of [Fe(CN)_6_]^4−/3−^ (*data not shown*). The values of the charge transfer resistance (*R*_*ct*_), Warburg impedance modulus (*σ*), and electrical double-layer differential capacitance (*C*_*dl*_) were determined by fitting the model spectra to experimental data, and on their basis the heterogeneous electrode reaction rate constant (*k*_*s*_) was determined (Table 1). The modification of SPCE with pp-AN nanofilm resulted in almost 3-fold decrease in the *R*_*ct*_ value, indicating both faster electron transfer kinetics and an expansion of the electrochemically active surface area, which resulted in 4-fold increase of electrode *k*_*s*_ as well as a decrease of *ΔE*. In addition, a *C*_*dl*_ decreased three times compared to bare SPCE, which translated in the reduction of capacitive component of current in CV measurements. Thus, it can be concluded that the pp-AN/SPCE is characterized by improved metrological parameters in comparison to SPCE.

**Table 1:** The comparison of SPCE and pp-AN/SPCE sensors based on parameters achieved from EIS measurements

Parameter	SPCE	pp-AN/SPCE
*R*_*ct*_/kΩ	12.0 ± 0.1	4.6 ± 0.1
*σ*/kΩ s^−1/2^	6.93 ± 0.02	11.3 ± 0.1
*C*_*dl*_/μF cm^−2^	636.6 ± 2.7	191.3 ± 1.6
*k*_*s*_/× 10^−5^ m s^−1^	(1.1 ± 0.1)	(4.3 ± 0.1)

### Research on the mechanism of the electrode reaction

**Fig. 4:**
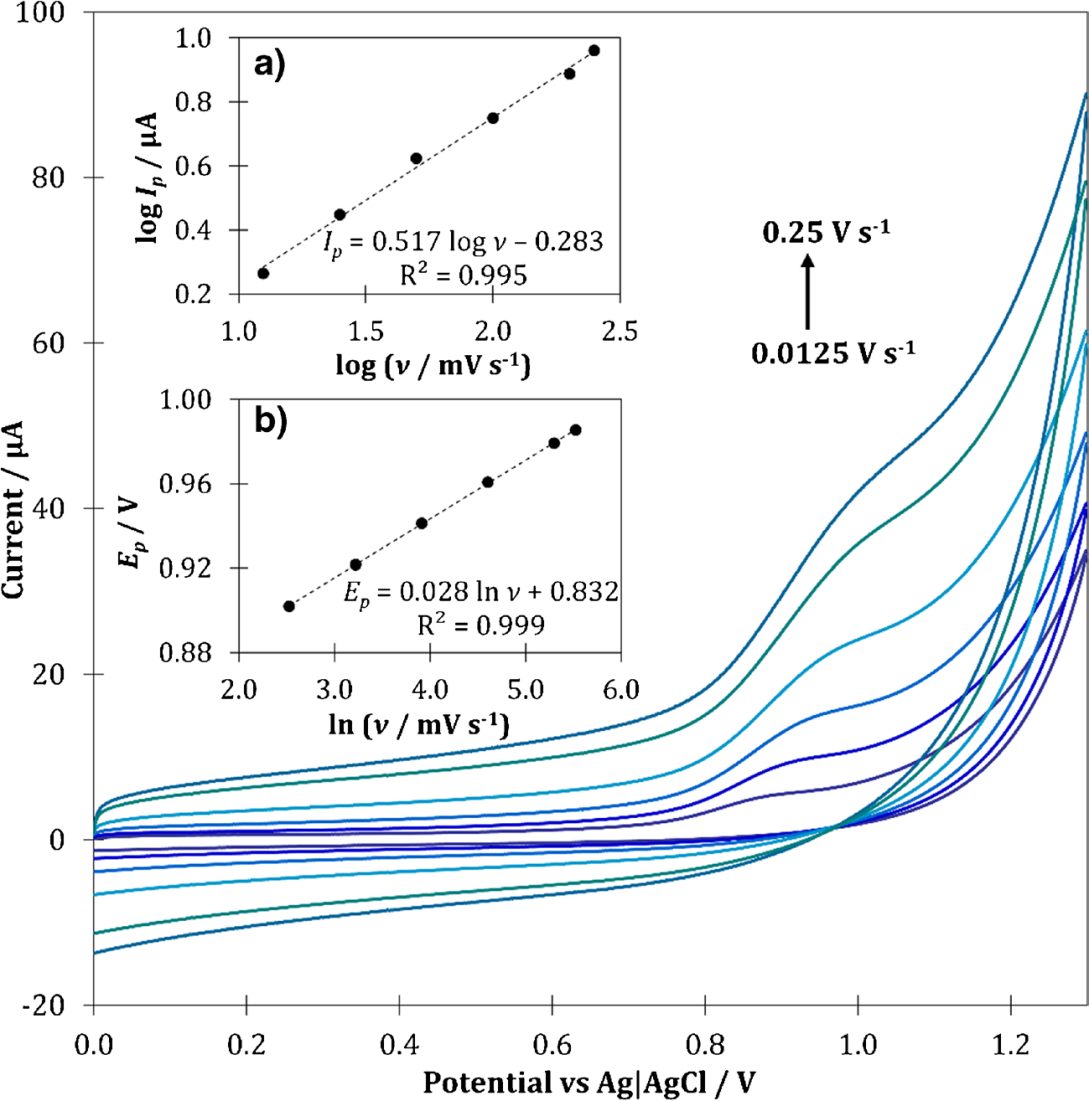
Cyclic voltammograms recorded using pp-AN/SPCE at different scan rates for 50 μmol L^−1^ BUP in 0.1 mol L^−1^ phosphate buffer with pH 7 with corresponding charts representing the influence of the scan rate on the current (**a**) and the potential (**b**) of the BUP oxidation peak

For a better understanding of the electron transport mechanism between BUP molecules and the surface of pp-AN/SPCE sensor, a series of CVs using various scan rates (*v*) was recorded (Fig. 4). The anodic peak current for 50 μmol L^−1^ of BUP increased successively with the scan rate from 0.0125 to 0.250 V s^−1^, and at the same time shifted towards more positive potential, which confirms the irreversibility of the electrode reaction [25]. The dependence of the log *I*_*p*_ vs log *v* was used for the verification of kinetic behavior of the reaction (Fig. 4a). The plot representing this relationship was characterized with good linearity with a slope close to theoretical value of 0.5, which indicates that BUP oxidation is a diffusion-controlled process [25].

Based on the change of peak potential (*E*_*p*_) resulting from the increasing scan rate (*v*), the number of electrons (*n*) involved in the electrode reaction was calculated. According to Laviron equation, for an irreversible, diffusion-controlled process, the relationship between the *E*_*p*_ and the ln *v* can be described by the following equation:

$${E}_p=A+\frac{RT}{nF}\mathit{\ln}\ v$$

where *A* is the constant value, *R* is the gas constant (*R* = 8314 J mol^−1^ K^−1^), *T* is the temperature, *F* is the Faraday constant (*F* = 96485 C mol^−1^) [26].

Based on recorded anodic voltammograms, the dependence of the *E*_*p*_ vs ln *v* was determined (Fig. 4b). The slope of the obtained linear relationship was equal to 0.028. Hence, the number of electrons involved in the electrode reaction was calculated as 0.914, indicating a participation of one electron in the BUP oxidation process.

**Fig. 5:**
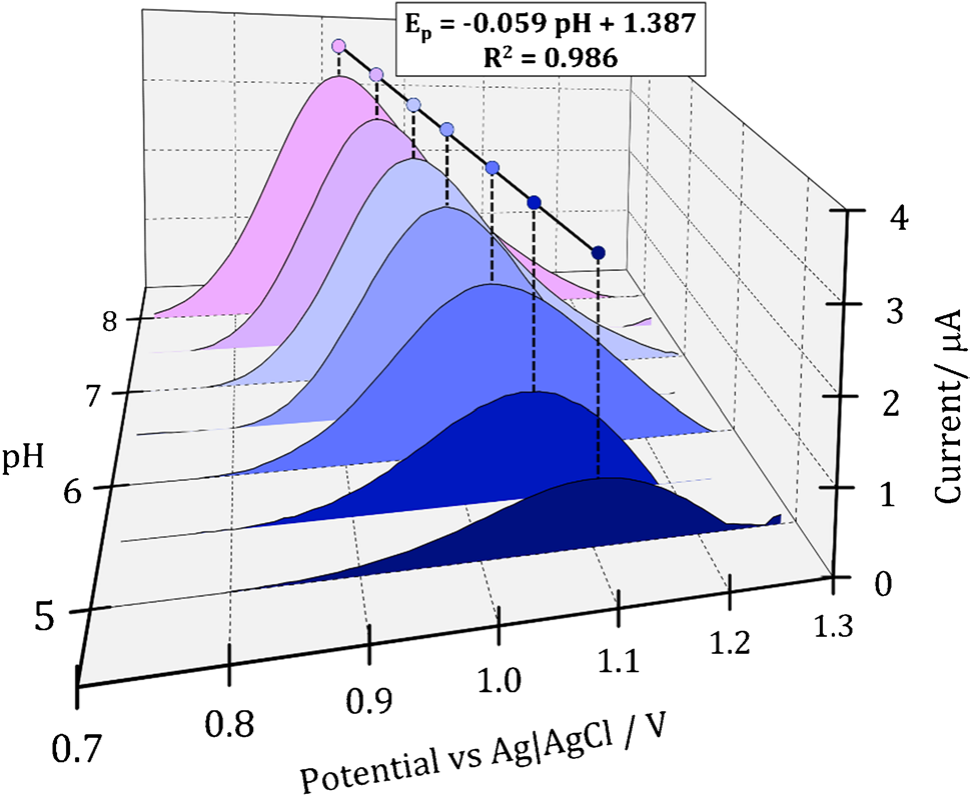
Cyclic voltammograms (after background subtraction) recorded using pp-AN/SPCE in buffer solutions of various pH (5–8) containing 50 μmol L^−1^ of BUP (*v* = 0.1 V s^−1^) including the dependence of peak potential on a buffer pH

In order to verify the participation of protons in the electrode reaction, the influence of buffer pH on the potential of the BUP oxidation peak was investigated. Therefore, a series of CVs was recorded for 50 μmol L^−1^ BUP in phosphate (pH 5–8) and ammonium (pH 8, 9, 10) buffers (Fig. 5). With the increase of pH from 5.0 to 8.0, the shift of the peak towards lower potential values was observed, which confirms the participation of protons in the electrode reaction. The plotted relationship between the peak potential and the pH of the electrolyte solution from 5.0 to 8.0 was linear with a Nernstian slope of −0.059 V pH^−1^, which indicates the participation of an equal number of electrons and protons in the BUP electrooxidation reaction [27]. For pH values above 8.0, the peak potential remained constant with increasing pH, which proves that the pH value corresponding to the pK_a_ of BUP was exceeded.

Moreover, the CV measurements conducted in various buffer solutions showed that the highest peak current was obtained in the ammonium buffer at pH 8.0. For lower pH (7.5–5.0), as well as for higher pH values (*data not shown*), the peak height gradually decreased. Thus, for further studies, ammonium buffer pH 8.0 with concentration of 0.1 mol L^−1^ was chosen.

Based on the obtained experimental results as well as literature reports on the electrooxidation of alkyl amine derivatives and ketones [28], it can be concluded that BUP undergoes an irreversible electro-oxidation reaction within the secondary amine group, which due to the loss of one electron and proton leads to the formation of an alkyl radical. The proposed mechanism of the electrode reaction is shown in Fig. S2.

### Analytical performance of pp-AN/SPCE

**Fig. 6:**
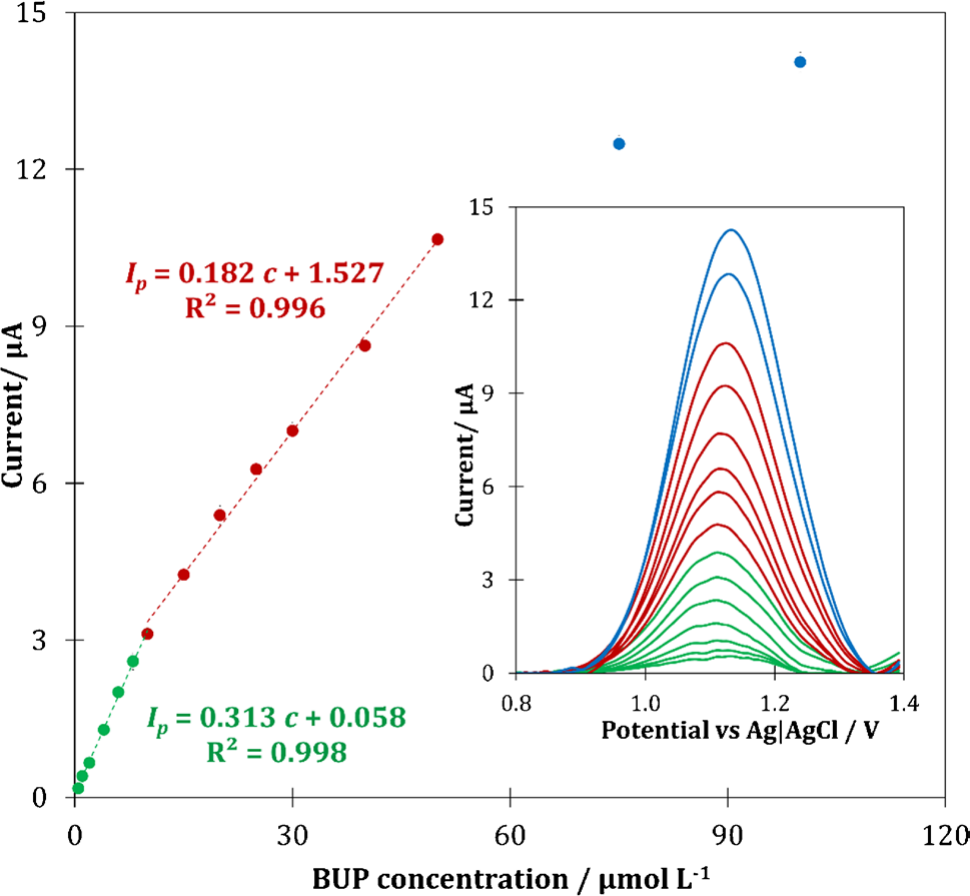
Calibration graph obtained on the basis of SCV voltammograms (insert) recorded for increasing concentration of BUP (0.63–100 μmol L^−1^) in 0.1 mol L^−1^ ammonium buffer pH 8 under optimal measurement conditions

The BUP quantification was performed using staircase voltammetry (SCV) in ammonium buffer pH 8 (0.1 mol L^−1^) at the following parameters: potential step of 8 mV, potential step of 80 ms, accumulation step at 100 mV for 120 s (*discussed in detail in the* *supplementary material*). Under the established optimal experimental conditions, the developed method of BUP determination was validated by determining parameters such as linearity range (LR), sensitivity, limit of detection (LOD), limit of quantification (LOQ), repeatability, reproducibility, and long-term stability. For this purpose, staircase voltammograms using pp-AN/SPCE sensor were recorded for increasing BUP concentrations in the range of 0.5–100 μmol L^−1^. From voltammograms subjected to background subtraction, the peak current values were acquired at potential ca. 1.1 V (vs. Ag|AgCl) and on their basis the calibration graph was prepared (Fig. 6).

The performed measurements for increasing concentration of BUP proved that the pp-AN/SPCE sensor showed a wide dynamic range, in which two linear ranges can be distinguished: 0.63–10.0 μmol L^−1^ and 10.0–50.0 μmol L^−1^, respectively. Based on the obtained director coefficient (*a*) of linear regression equation (Fig. 6) and previously determined surface area of the sensor (*A*_*el*_ = 0.131 cm^2^), the sensitivity was calculated, and for lower LR it was 2.39 ± 0.19 μA L μmol^−1^ cm^−2^ and for higher LR it was 1.35 ± 0.19 μA L μmol^−1^ cm^−2^. Moreover, on the data achieved for lower LR, the LOD and LOQ values were assessed according to the formulas *LOD = 3.3 SD / a* and *LOQ = 3 LOD*, where SD is a standard deviation of analytical signal obtained for the lowest tested concentration of BUP solution (*n* = 5) [29]*.* The calculated LOD and LOQ values were 0.21 μmol L^−1^ and 0.63 μmol L^−1^, respectively. Additionally, based on the sensitivity determined for the lower LR, the repeatability and reproducibility of the method were determined. For three series of measurements (*n* = 3), conducted at the same sensor, the repeatability of 5.5% was obtained, while for independent series of measurements carried out with the use of different pp-AN/SPCEs (*n* = 8) obtained from one PECVD process, the reproducibility was 7.1%. Additionally, for independent series of measurements carried out using eight sensors, two sensors each from four PECVD processes (conducted at different time but under the same conditions), the reproducibility was 12%. In order to assess a long-term stability of the method, the variation in the sensitivity of measurements carried out with one sensor for 30 days was examined. During the tested time period, a systematic slight decrease in sensitivity was observed, which after 30 days reached a value close to 18%, which indicates the satisfactory stability of the pp-AN/SPCE sensor. Analogous stability tests were performed using five independent sensors. For all tested pp-AN/SPCEs, a proportional decline in sensitivity was also observed, which reached a 13% decrease after 21 days. Conducted long-term stability tests indicate satisfactory stability of the pp-AN/SPCE sensors. Interestingly, three pp-AN/SPCEs tested for 0.2 mmol L^−1^ of model redox probe [Fe(CN)_6_]^4−/3−^ retained their characteristic parameters after 6 months of storage in the laboratory atmosphere, while the sensitivity of bare SPCEs, kept under the same conditions, dropped by a few percent during this time and Δ*E* increased by a few millivolts. The robustness of the developed sensor should be considered satisfactory. Based on the optimization experiments performed, it can be concluded that a slight change in the parameters of analysis, including the pH and concentration of the buffer used as a supporting electrolyte, as well as the SCV parameters, should not significantly affect the analytical performance of the developed sensor. Moreover, the interference tests proved the good selectivity of developed sensor (*discussed in the* *supplementary material*).

Compared to previously reported electrochemical methods of BUP determination (Tab. S3), fabricated sensor exhibits almost the widest linear range among all sensors described in literature so far (except ISEs [13]). Although the pp-AN/SPCE sensor is less sensitive than mercury electrode [14], it is worth noting that these electrodes have already been withdrawn from the use due to the toxicity of mercury vapor. The GCE [15] shows similar analytical parameters in terms of BUP determination as the mass-produced pp-AN/SPCE, but it is expensive and cannot be adopted in complex measurement systems (e.g., flow-through) offered by Methrom. Admittedly, the use of EIS technique allows to obtain very low LOD [16]; however, it also requires expensive equipment and the ability to use specialized software as well as many years of experience in interpreting EIS spectra. Importantly, the developed method is the first published so far, which is based on the electrooxidation of the secondary amine group of BUP. In other works, the recorded signal is related to the reduction of the carbonyl group present in the BUP molecule, which corresponds to the cathodic peak at potential ca. −1.2 V [15].

### Real sample analysis

For the verification of utility of the pp-AN/SPCEs in the analysis of real samples, attempts were made to determine BUP in environmental and biological samples enriched with analyte. For that purpose, the Vistula river water and CRMs of wastewater and groundwater were spiked with BUP standard. For each sample, analysis was conducted three times (*in accordance with the procedure described in chapter 2.5*) and then recovery and relative standard deviation (RSD) were determined to assess accuracy and precision of the method (Table 2).

**Table 2:** Results of BUP determination in samples enriched with analyte

Sample	Addition of BUP/*μmol L*^*-1*^	Concentration determined/*μmol L*^−*1*^	Mean concentration* ± CI/*μmol L*^−*1*^	Recovery*/*%*	RSD/*%*
Vistula river water^1^	7.50	7.08 7.69 7.41	7.39 ± 0.51	98.6	4.1
Wastewater CRM^1^	37.5	37.6 37.8 36.8	37.4 ± 0.9	99.7	1.4
Groundwater CRM^1^	37.5	36.6 36.4 35.2	36.1 ± 1.3	96.2	2.1
Urine^2^	250	250 250 259	253 ± 8	102	2.0
Blood serum^2^	1000	1009 973 997	993 ± 31	99.9	1.8

*Mean value (*n* = 3)

^1^Result obtained with CSAM

^2^Results achieved with SISAM

For Vistula water as well as CRMs of wastewater and groundwater, a satisfactory recovery, ranging from 96.2 to 99.7%, and acceptable RSD (not higher than 4.1%) were obtained. Thus, it can be concluded that the developed sensor enables the determination of BUP in environmental samples with good accuracy and precision.

The usefulness of the developed method was also examined by analyzing samples with a more complex matrix, i.e., synthetic urine and blood serum spiked with the addition of the BUP standard. Preliminary research has shown that high dilution rate of biological matrix is not sufficient to obtain satisfactory accuracy and precision of analysis. In case of both urine and serum, recovery values reached 120% and RSD was ca. 20%. In order to minimize observed interferences, instead of the standard addition method, the modified version of this calibration approach was used, i.e., the signal increment standard addition method (SISAM). This method enables to increase the accuracy of the analysis by extrapolating the calibration plot to the signal measured for standard solution (added to the measurement vessel before sample), and not to the background signal, as in the case of the conventional standard addition method. The principles of SISAM calibration method were described in detail in this work [30]. On the basis of the obtained results using SISAM method, the recovery and RSD values were determined (Table 2). The use of new calibration approach (SISAM) made it possible to determine BUP with high accuracy and precision, which was confirmed by the obtained recovery values (99.9–102%) and RSD below 2%. Overall, the results of the analysis of water samples and biological materials show that the developed pp-AN/SPCE sensor can find practical application for the determination of BUP even in the presence of complex matrix.

## Conclusions

The use of PECVD technology as a method of depositing a functionalized polymer or nanocomposite layer on SPEs enables modification of the electrochemical properties of the working electrode, while maintaining repeatability and reproducibility of its metrological and functional parameters. What is particularly important, the PECVD technology provides the possibility of standardization of the produced sensors, which is not possible in the case of most concepts proposed in the literature. Different composition of precursors and conditions of the PECVD process enable the deposition of layers with different topography, physical and chemical properties, thickness, and electrochemical characteristics. It is worth pointing out that the described new original method of SPEs modification is not designed to the determination of a specific analyte or group of analytes. It can be tested for the analysis of any analyte(s) that undergoes redox transformations in the range of pp-AN/SPCE working potentials (−0.4–1.3 V). The developed sensor showed specific selectivity and sensitivity towards bupropion (BUP), an antidepressant drug widely used since the outbreak of the COVID-19 pandemic. The proposed method of BUP detection, which does not require preliminary preparation of the sample for analysis (in contrast to chromatographic methods), has been validated and successfully used for the determination of BUP in environmental samples of river water, groundwater, and sewage, as well as synthetic urine and blood serum. The pp-AN/SPCE sensor, despite high sensitivity towards BUP detection, satisfactory selectivity, and outstanding stability, also has some limitations. Its main disadvantage is the narrow range of linearity, which makes it difficult to apply this procedure in laboratory analysis. As a result, the preparation of the analytical sample may require further dilutions so that the signal from the sample and subsequent additions of the standard is within the appropriate range. Although the pp-AN/SPAuE sensor did not show electrochemical sensitivity to BUP, its specific features characteristic for a microelectrode array make it suitable for other applications, such as flow systems or environmental monitoring.

### Supplementary information

ESM 1
