# Supplementary material for: Electrochemical sensing platform based on screen-printed carbon electrode modified with plasma polymerized acrylonitrile nanofilms for determination of bupropion
Source: Mikrochim Acta. 2023 Sep 14;190(10):391. doi: 10.1007/s00604-023-05971-0 (PMC10499721; doi:10.1007/s00604-023-05971-0)
Supplement: Supplementary file 1 — ESM 1 [file 604_2023_5971_MOESM1_ESM.docx]

**SUPPORTING INFORMATION**

**Electrochemical sensing platform based on screen-printed carbon electrode modified with plasma polymerized acrylonitrile nanofilms for determination of bupropion**

Maria Madej^1,*^, Agata Trzcińska^1^, Justyna Lipińska^2^, Ryszard Kapica^3^, Maciej Fronczak^3^, Radosław Porada^1^, Jolanta Kochana^1^, Bogusław Baś^2^, Jacek Tyczkowski^3^

**^1^** Jagiellonian University, Faculty of Chemistry, Department of Analytical Chemistry, Gronostajowa 2, 30-387, Kraków, Poland

^2^ AGH University of Science and Technology, Faculty of Materials and Ceramics, Department of Analytical Chemistry and Biochemistry, A. Mickiewicza 30, 30-059, Kraków, Poland

^3^ Lodz University of Technology, Faculty of Process and Environmental Engineering, Department of Molecular Engineering, Wólczańska 213, 93-005, Lodz, Poland

* **Corresponding author:** e-mail: marysia.madej@uj.edu.pl; https://orcid.org/0000-0002-7979-2922

**
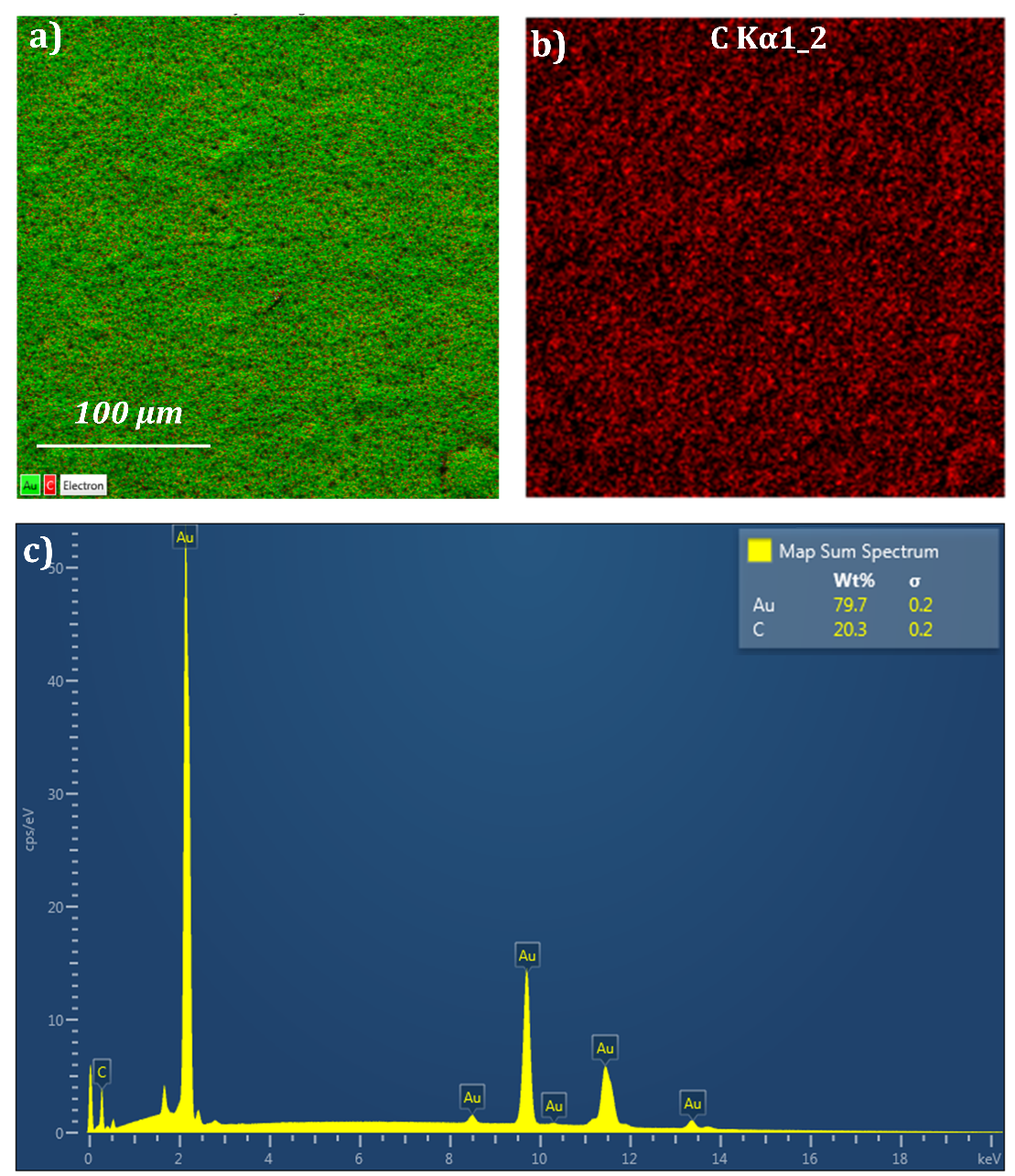
**

**Fig. S1** *EDS mapping* *of the surface of pp-AN/SPAuE (a) with detailing the carbon distribution (b) and spectrum representing the mass distribution of elements at gold electrode surface.*

**Tab. S1** *Summary of elemental analysis of the SPCE and pp-AN/SPCE sensors using X-ray photoelectron spectroscopy (XPS) and energy dispersive spectroscopy (EDS) (n = 3).*

| **Element** | | **Mass concentration ± SD / %** | | |
| --- | --- | --- | --- | --- |
|  |  | **SPCE** | **pp-AN/SPCE** | |
| ***XPS*** | | | | |
| O | 10.77 ± 1.27 | | | 15.39 ± 0.98 |
| N | 0.00 ± 0.00 | | | 13.32 ± 1.15 |
| C | 89.23 ± 0.92 | | | 71.29 ± 1.61 |
| ***EDS*** | | | | |
| O | 2.6 ± 0.3 | | | 5.1 ± 0.6 |
| N | 0.0 ± 0.0 | | | 9.4 ± 1.2 |
| C | 97.4 ± 0.3 | | | 85.5 ± 1.2 |

**XPS spectra analysis**

To more precisely determine changes in the molecular structure of the SPCE surface after pp-AN deposition, the narrow XPS spectra for the regions corresponding to C1s (282 – 291 eV) and N1s (395 – 405 eV) were analyzed in detail, according to the literature [17–20] (Fig. 2). The C1s SPCE spectrum can be deconvoluted into four bands labeled C1 (284.6 eV), C2 (285.4 eV), C3 (286.6 eV), and C5 (289.0 eV), while the C1s pp-AN/SPCE spectrum contains one additional band labeled C4 (287.7 eV). In turn, in the N1s spectrum for the SPCE bare electrode, as expected, we do not see any bands, while in the pp-AN/SPCE spectrum, we can distinguish two bands: N1 (399.5 eV) and N2 (401.5 eV). The C1 band was used as a reference band to calibrate all spectra. This band is attributed to C=C bonds, which in the SPCE are characteristic of the graphitic structure (sp2 carbon). The presence of this band in pp-AN reveals that the plasma-deposited film also contains sp2 carbon. Taking into account the structure of polyacrylonitrile synthesized by typical polymerization and its subsequent pyrolysis [21, 22], the sp2 carbon should be considered characteristic of heterocyclic structures related to pyridine. The presence of pyridine rings in pp-AN is also confirmed by the N1 band in the N1s spectrum. However, this band can also be attributed to nitrile groups derived from acrylonitrile molecules, which would suggest the presence of fragments of polyacrylonitrile chains in the film, as well as to conjugated imines formed during the cyclization of the nitrile groups. The possibility of such structures in the pp-AN film is confirmed by the sp3 carbon in the C–C and C–H bonds (C2 band). On the other hand, the N2 band, which is attributed to graphitic nitrogen, indicates significantly more advanced condensation in the film structure and the presence of graphite-like domains.

Continuing the analysis of the C1s spectrum, we can conclude that the location of the C3 band indicates the presence of C–O (C–OH) groups in both the SPCE and pp-AN, with C=N bonds also assigned here in the latter case. The presence of C=N groups confirms the observations presented above that the pp-AN film deposited from acrylonitrile under the applied PECVD conditions contains cyclic structures with nitrogen, resulting from the reconstruction of vinyl and nitrile functional groups. This result is complemented by the presence of the C4 band, to which, apart from the typical C=O bond, also H–N–C=N and possibly N–C=O moieties can be assigned. This clearly demonstrates the plasma fragmentation of the precursor molecules, which is responsible for the formation of a cross-linked structure that deviates from the "classic" ordered structure of thermally pyrolyzed polyacrylonitrile. The last band in the C1s spectrum, C5, is attributed to the more developed combinations of oxygen and carbon, such as R−O−C=O. In the pp-AN film, N–C(N)–N and N–C(O)–N can also be expected at this location.


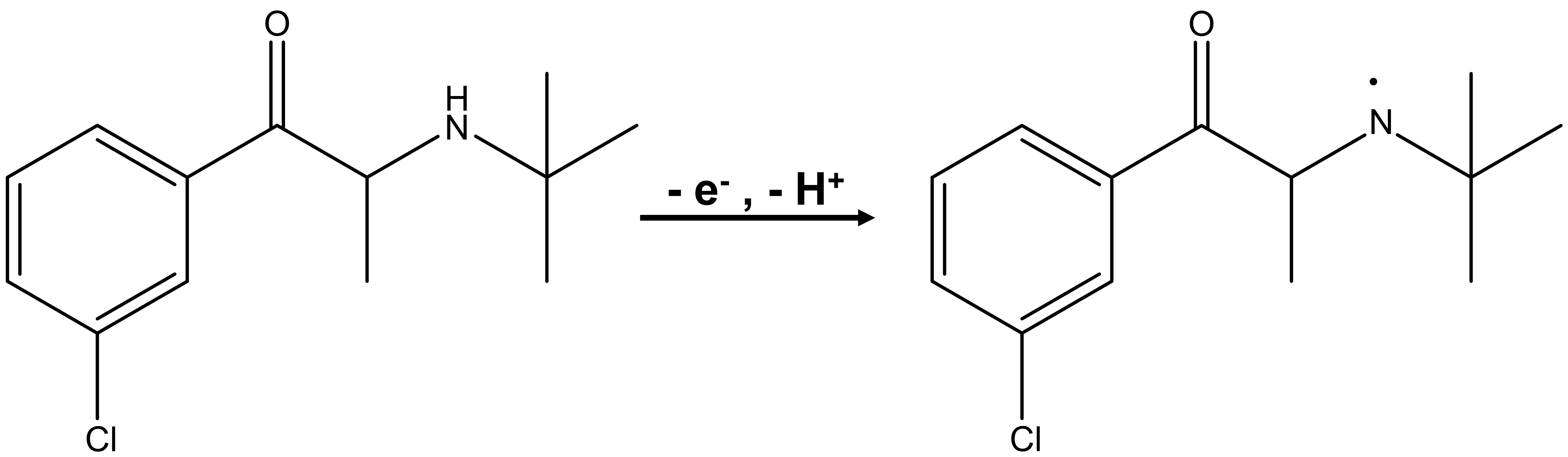


**Fig. S2** *Proposed reaction mechanism of bupropion electrooxidation on pp-AN/SPCE electrode.*

**Optimization of the measurement conditions**

To ensure the most favorable conditions for the BUP determination with the use of the pp-AN/SPCE sensor the composition and concentration of the supporting electrolyte was optimized as well as type of voltammetric technique and its instrumental parameters were chosen. The cyclic voltammograms recorded in various buffer solutions (*presented at Fig. 5 in the Manuscript*) proved that the highest and the best formed BUP oxidation peak is obtained in 0.1 mol L^-1^ ammonium buffer solution at pH 8, therefore this solution was chosen as the optimal supporting electrolyte.

Voltammograms recorded for 50 µmol L^-1^ of BUP in the presence of ammonium buffer pH 8 with different concentrations (Fig. S3) showed that the best conditions for BUP oxidation are provided by a buffer with a concentration of 0.1 mol L^-1^. The increase of buffer concentration not only negatively affected the peak height, but also caused the shift of peak potential towards more positive values. Additionally, an increase of the buffer concentration resulted in a growth of the background current, which is an undesirable effect due to decrease of the method sensitivity. Also, for lower concentration of ammonium buffer i.e. 0.05 mol L^-1^, a slight decrease of peak current was observed. Thus, 0.1 mol L^-1^ ammonium buffer at pH 8 was selected for further research.


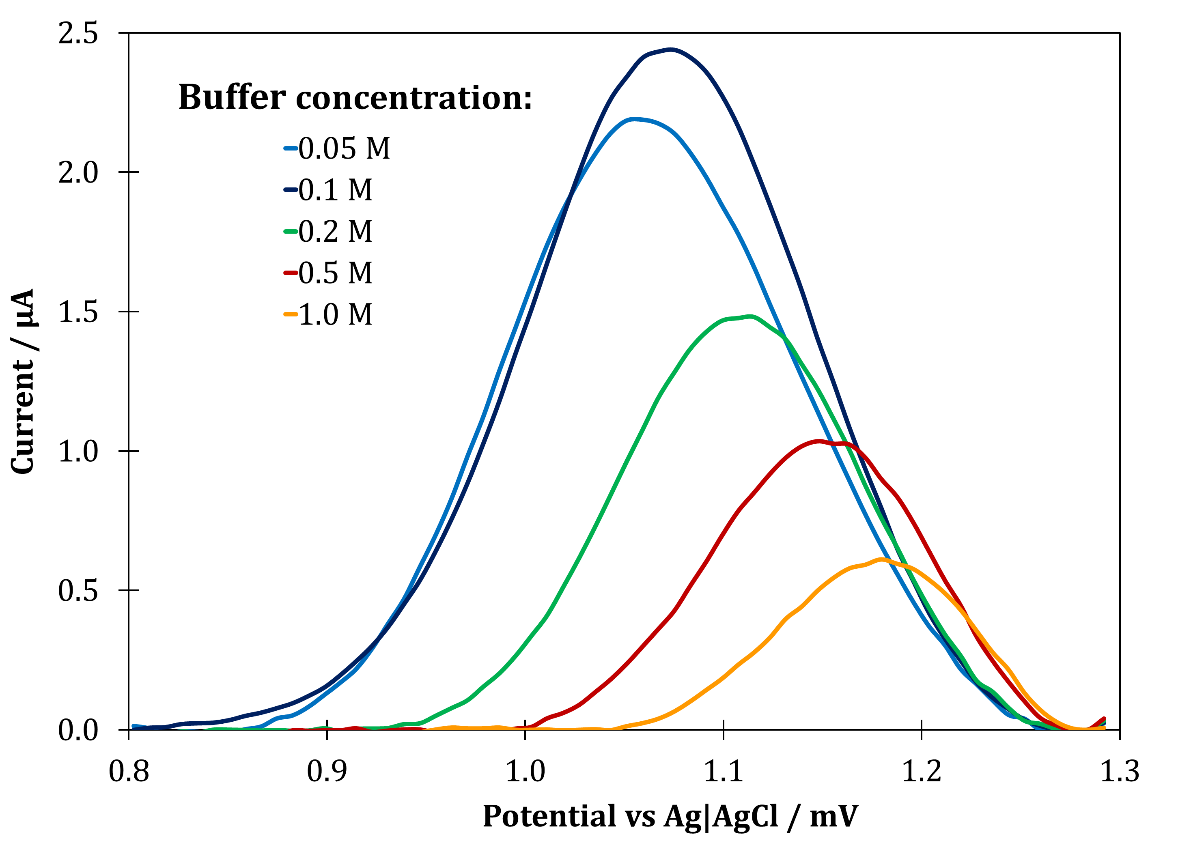


**Fig. S3** *Influence of ammonium buffer pH 8 concentration on the height of the BUP oxidation peak (voltammograms subjected to background subtraction).*

In the next step, the measurement technique was selected by comparing the shape, height and S/N ratio of BUP oxidation peaks obtained using cyclic (CV), staircase (SCV) and pulse differential voltammetry (DPV). In measurements carried out using the CV technique, due to the linear change of potential, the capacitive component of the current dominates. Therefore, CV measurements are characterized by lower sensitivity in comparison to SCV technique, for which the step change of potential with current measurement at the end of the step enables effective elimination of the capacitive component of the measured current. Interestingly, in the case of the DPV technique, a 13-fold decrease in the intensity of the peak current was observed, compared to the SCV technique. This is justified by the high differential capacitance of the electrical double layer of the pp-AN/SPCE sensor, as well as, the specificity of rapid impulse changes of the electrode polarization potential and the extension of the recording time of the DPV curve. Due to the fact that the highest sensitivity of BUP determination as well as the most favorable S/N ratio were obtained by SCV, therefore this technique was chosen for further research.

Consecutively, the instrumental parameters of the SCV technique were optimized, i.e. potential step (*E_s_*) and its width (*t_s_*), maintaining constant accumulation potential (0 V) and time (30 s). The peak current intensity, the S/N ratio and the repeatability of the recorded voltammograms were considered as optimization criteria. The tested parameters and the values indicated as optimal are presented at Fig. S4 a, b. As can be seen, the well-defined peak current with the best repeatability was obtained for potential step of 8 mV and pulse width of 80 ms. In order to increase the sensitivity of the method the potential (*E_acc_*) and the accumulation time (*t_acc_*) were also optimized (Fig. S4 c, d). The extension of a preconcentration step allowed for an approx. 20% increase in the BUP oxidation signal. As optimal values an accumulation potential of 100 mV and accumulation times of 120 s were chosen.


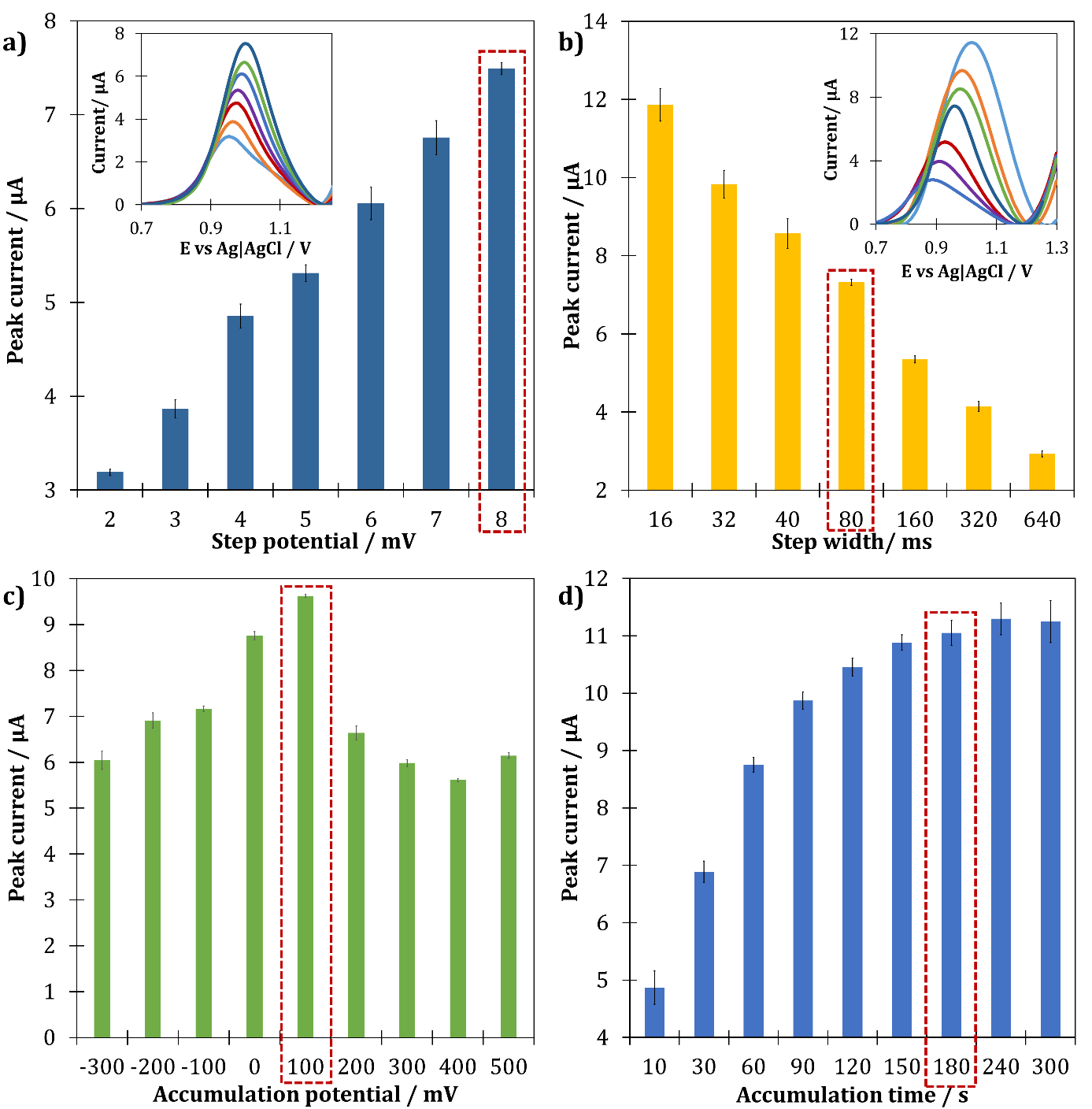

**Fig. S4** *Influence of staircase voltammetry (SCV) parameters: step potential (a), step width (b), accumulation potential (c) and accumulation time (d) on the BUP oxidation peak current (50 µmol L^-1^); the parameters chosen as optimal values marked with red, dashed line.*

**Interference studies**

As a part of analytical characteristics of the developed method, the ability of pp-AN/SPCE sensor for the determination of BUP in the presence of interfering species was evaluated. For that purpose, a variety of organic and inorganic compounds (in concentration between 10 – 500 µmol L^-1^), such as ions and compounds commonly occurring in environmental waters i.e. Mg^2+^, Na^+^, K^+^, Cl^-^, NO_3_^-^, SO_4_^2-^, humic acid, Triton X-100 (non-ionic surfactant), sodium dodecyl sulphate (SDS; anionic surfactant), hexadecyltrimethylammonium bromide (CTAB; cationic surfactant), as well as substances used as fillers in pharmaceutical products (talc, TiO_2_, glucose, lactose, starch, magnesium stearate) were tested.

Performed measurements confirmed that the majority of tested compounds, regardless of their concentration, did not cause a significant change in the height of peak recorded for 50 µmol L^-1^ of BUP (less than 10%), as well as did not affect its shape and position (Tab. S3). Only the presence of surfactants had a distinct influence on the recorded signal. In the case of Triton X-100 the measured peak current for BUP increased even twice. Whereas, in the presence of CTAB analytical signal decreased by up to 44%. Hence, during the analysis of highly polluted environmental samples, the risk of undesirable interference from surfactants (especially non-ionic and cationic) should be considered.

Considering the potential use of the fabricated sensor for the biological samples analysis, the influence of synthetic certified reference materials (CRMs) of urine and blood serum on the recorded signal for BUP was also assessed (Fig. S5). The conducted tests showed that for the biological matrix concentration in the ammonium buffer below 0.5% vol. the signal drop is negligible. However, with higher content of urine or blood serum the recorded peak current drastically decrease, reaching 80% signal drop for 4.0% vol. of biological matrix content. The obtained results indicate that the pp-AN/SPCE sensor can be used for the determination of BUP in biological samples, however, a sufficiently high dilution of the sample (at least 100-fold) is necessary.

The conducted interference tests indicate a satisfactory selectivity of the developed sensor. The unfavorable interferences related to the presence of surfactants and the biological matrix can be reduced by implementation of extraction at the stage of sample preparation for analysis or, in less conventional way, by the use of an appropriate calibration method which could compensate the interference effects [26].

**Tab. S2** *Verification of interference effects of different inorganic and organic compounds on the oxidation peak current recorded for 50 µmol L^-1^ concentration of BUP.*

| Tested interferent | Signal change (*%*) caused by the presence of an interferent in concentration / *µmol L^-1^* * | | | | | |
| --- | --- | --- | --- | --- | --- | --- |
|  | **10** | **25** | **50** | **100** | **200** | **500** |
| Inorganic compounds | | | | | | |
| KCl | -1.7 | -0.8 | -2.8 | -2.1 | -2.0 | -3.4 |
| NaNO_3_ | -2.2 | -3.7 | -4.8 | -7.7 | -8.4 | -8.4 |
| MgSO_4_ | -6.4 | -3.1 | -3.2 | -5.3 | -7.5 | -7.2 |
| TiO_2_ | -2.2 | -3.6 | -2.9 | -3.3 | -4.7 | -7.5 |
| Talc | -1.8 | -2.6 | -6.3 | -5.2 | -8.7 | -8.6 |
| Organic compounds | | | | | | |
| Glucose | -3.2 | -3.4 | -3.5 | -4.4 | -4.7 | -5.7 |
| Lactose | -2.7 | -2.9 | -2.8 | -4.0 | -4.2 | -5.3 |
| Starch | -2.4 | -0.1 | -4.4 | -7.3 | -5.6 | -7.1 |
| Magnesium stearate | -0.9 | -1.6 | -5.5 | -4.9 | -7.9 | -8.2 |
| Humic acid | -0.4 | -0.5 | -2.0 | -3.4 | -4.1 | -5.6 |
| Surfactants | | | | | | |
| Triton X-100 | 4.8 | 7.2 | **71.7** | **108.4** | **112.5** | **71.5** |
| SDS | -0.5 | -3.1 | -3.3 | 7.7 | 9.4 | **18.6** |
| CTAB | -2.1 | **-13.0** | **-34.4** | **-44.0** | **-29.1** | **-** |

* negative values refer to the decrease in the oxidation peak current caused by the presence of an interferent, whereas a positive values indicate an increase in the obtained signal.


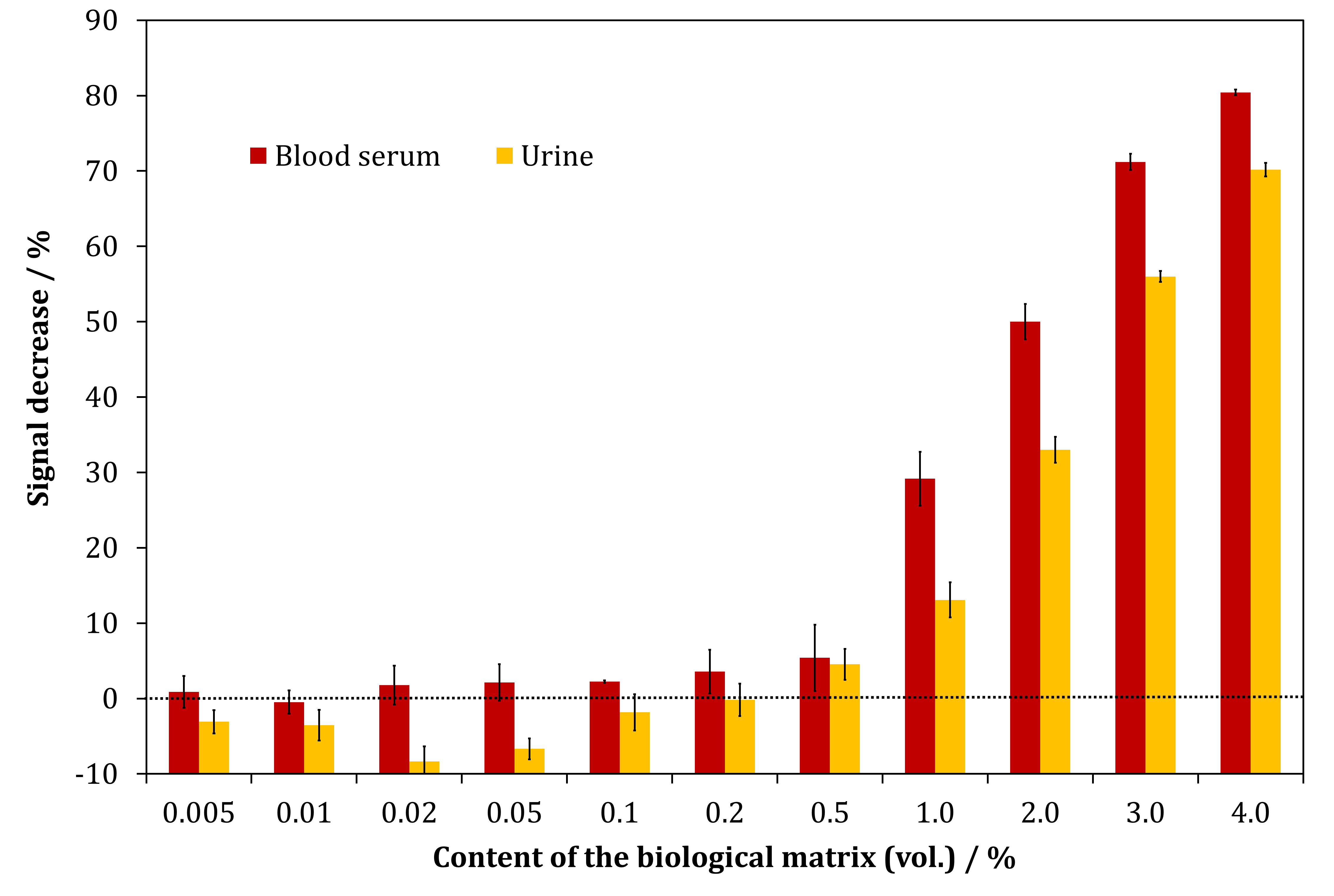


**Fig. S5** *Signal changes caused by the occurrence of increasing content of synthetic urine (yellow) and blood serum (red) in ammonium buffer (pH 8; 0.1 mol L^-1^) containing 50 µmol L^-1^ of BUP; SCV parameters: E_s_ = 8 mV; t_s_ = 80 ms, E_acc_ = 100 mV, t_acc_ = 120 s.*

**Tab. S3** *Comparison of BUP determination methods using different sensors and electrochemical methods.*

| **Electrode** | **Technique** | **Linear range / *µmol L^-1^*** | **LOD**  **/ *µmol L^-1^*** | **Samples** | **Ref.** |
| --- | --- | --- | --- | --- | --- |
| PCV/BUP-STB^1^  BUP-STB-CILE^2^ | Potentiometry | 10 – 10 000  5 – 10 000 | 8.5  3.1 | Tablets | [13] |
| DME^3^ | Polarography | 0.005 – 0.5 | 0.001 | Urine | [14] |
| GCE | SWCAdSV^4^ | 1.0 – 5.6 | 0.13 | Tablets | [15] |
| MIP/AuNPs/GO/SPCE^5^ | EIS | 0.002 – 0.99 | 0.001 | Tablets, urine | [16] |
| pp-AN/SPCE | SCV | 0.63 – 10.0  10.0 – 50.0 | 0.21 | Water, urine, blood serum | This work |

^1^ ion-selective electrode with a poly(vinyl chloride) membrane and on pair BUP-sodium tetraphenylborate, ^2^ paste electrode based on an ionic liquid obtained by mixing graphite powder, multi-wall carbon nanotubes, 1-n-butyl-3-methylimidazolium tetrafluoroborate and BUP ion pair BUP-sodium tetraphenylborate, ^3^ drop mercury electrode, ^4^ square wave cathodic adsorption stripping voltammetry, ^5^ carbon screen-printed electrode modified with molecular imprinted polymer, gold nanoparticles and graphene oxide
